# Respiratory Flora Intervention: A New Strategy for the Prevention and Treatment of Occupationally Related Respiratory Allergy in Healthcare Workers

**DOI:** 10.3390/microorganisms12122653

**Published:** 2024-12-20

**Authors:** Linglin Gao, Xi Chen, Ziyi Jiang, Jie Zhu, Qiang Wang

**Affiliations:** Hubei Province Key Laboratory of Occupational Hazard Identification and Control, Institute of Infection, Immunology and Tumor Microenvironment, Medical College, Wuhan University of Science and Technology, Wuhan 430065, China; gaolinglin@wust.edu.cn (L.G.); chenxi001009@163.com (X.C.); jiangziyi@wust.edu.cn (Z.J.); 17703698702@163.com (J.Z.)

**Keywords:** flora interventions, healthcare workers, occupational exposure, occupational allergic respiratory diseases

## Abstract

Occupational allergic respiratory disease in healthcare workers due to occupational exposure has received widespread attention. At the same time, evidence of altered respiratory flora associated with the development of allergy has been found in relevant epidemiologic studies. It is of concern that the composition of nasopharyngeal flora in healthcare workers differs significantly from that of non-healthcare workers due to occupational factors, with a particularly high prevalence of carriage of pathogenic and drug-resistant bacteria. Recent studies have found that interventions with upper respiratory tract probiotics can significantly reduce the incidence of respiratory allergies and infections. We searched PubMed and other databases to describe the burden of allergic respiratory disease and altered respiratory flora in healthcare workers in this narrative review, and we summarize the mechanisms and current state of clinical research on the use of flora interventions to ameliorate respiratory allergy, with the aim of providing a new direction for protecting the respiratory health of healthcare workers.

## 1. Introduction

During the COVID-19 pandemic, the health and safety of frontline healthcare workers were at great risk [1,2,3], and according to an analysis of 97 studies published in 2020, the prevalence of SARS-CoV-2 infection among healthcare workers using RT-PCR screening was estimated to be 11%, with nurses being the most commonly affected [4]. SARS-CoV-2 causes mild to severe respiratory infectious disease in humans [5].

Most people, not only healthcare workers, are affected by a multitude of respiratory diseases in their daily lives and work; in particular, chronic respiratory diseases due to occupational exposure have become the most common occupational disease [6]. Among them, allergic rhinitis affects more than 400 million people globally in their daily life and work–study [7], and is an upper respiratory tract disease with a high prevalence around the world [8]. Occupational allergens are a risk factor for the development of allergic rhinitis [9]. Occupational allergens for healthcare workers include detergents and disinfectants, natural rubber, latex, and a variety of drugs and reagents, and healthcare workers exposed to these occupational allergens are at risk of occupationally related rhinitis. There are more than 339 million asthma sufferers worldwide, with asthma prevalence rates as high as 18% in some countries [10]. Asthma involves the interaction of genetic, immune, and environmental factors [11,12]. Exposure to the workplace significantly increases the prevalence of chronic respiratory diseases such as asthma [13]. However, long-term use of common medications for asthma relief, such as inhaled corticosteroids [14], suppresses the intrinsic immunomodulatory pathways of the respiratory mucosa, enhances viral replication, and reduces host resistance to respiratory viral infections, while increased mucus production exacerbates symptoms. Experts have also found that inhaled corticosteroids increase the chance of opportunistic pathogens adhering to and colonizing the mucous membranes of the respiratory tract, thereby increasing the risk of respiratory tract infections and diseases [15]. Therefore, we believe that enhancing the intrinsic immune regulatory mechanisms of the respiratory mucosa is an effective strategy to avoid the side effects of long-term reliance on drug therapy.

**Figure 1:**
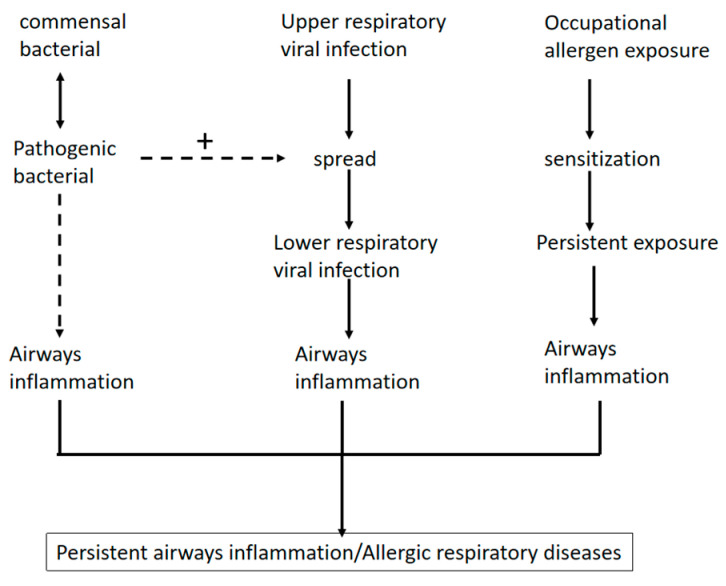
Dysbiosis of the nasal flora can induce allergic respiratory diseases. In dysbiosis, pathogenic bacteria cause airway inflammation and, together with respiratory viruses, lower respiratory tract infections. Occupational allergen exposure increases the body’s susceptibility to pathogens, and continued exposure can lead to airway inflammation or even induce respiratory allergies.

In addition to the occupational allergens mentioned above, the adhesion and colonization of nasal mucosa by opportunistic pathogens such as *Staphylococcus*, *Escherichia coli* (*E. coli*), *Klebsiella*, *Enterobacter*, and *Salmonella* present in the daily working environment of healthcare workers is also a major factor in triggering chronic airway inflammation and thus allergic respiratory diseases [16,17]. There have been reports that healthcare workers’ clothing harbors a wide range of multi-drug-resistant bacteria that can be transmitted to patients [18]. Opportunistic pathogens are detected in the oral cavity of healthcare workers at a higher rate than non-healthcare workers. They are more pathogenic and resistant, increasing the risk of transmission of opportunistic pathogens from healthcare workers to their families or partners. It has been shown that occupational exposures lead to alterations in the diversity and abundance of an individual’s microbiota, resulting in a variety of acute and chronic diseases, and that alterations in the microbiota play an important role, particularly in the development of many inflammatory diseases (Figure 1). It has been demonstrated that the composition of the nasal microbiota of patients with asthma and allergic rhinitis differs significantly from that of healthy controls and that the abundance and homogeneity of the nasal microbiota are strongly associated with a poor prognosis in asthma [19].

Exposure to certain allergens and bacteria early in life is beneficial in preventing allergic asthma [20]. Germ-free animals show impaired immune function, atrophy of lymphoid organs, and reduced production of immunoglobulin A (IgA), and are therefore more susceptible to infections and allergies [21]. The bacterial genera *Lachnospira*, *Veillonella*, *Faecalibacterium*, and *Rothia* in the gut microbiota of children at risk of asthma were significantly lower than that of healthy children, whereas inoculation of germ-free mice with these four species of bacteria resulted in improved airway inflammation in the adult offspring of the mice [22]. This suggests that these bacteria play an important role in preventing the development of asthma and that in the future it may be possible to prevent and treat asthma, as well as other respiratory allergic diseases, by means of probiotics. Accordingly, this narrative review is focused on the progress of research on the use of probiotics for the prevention and treatment of respiratory allergies in healthcare workers.

## 2. Methods

This narrative review is divided into three steps: searching the literature related to the topic, reviewing the abstract and full text, and discussing the results. Therefore, PubMed, Web of Science, Science Direct, and Google Scholar databases were searched to select relevant studies based on the topic of the review. The search was conducted in September 2023 and primarily searched for English-language articles published in major journals. The keyword “flora intervention” was used in conjunction with other terms such as healthcare workers, occupational exposure, respiratory sensitization, respiratory infections, respiratory flora, burden of disease, flora balance, and probiotics. After completing the search, abstracts were read to ensure that they addressed the topic of the review. All duplicates were removed, and the abstracts of the remaining articles were reviewed to ensure they met the inclusion criteria for the review. Inclusion criteria were studies that analyzed healthcare workers from at least one of three aspects: differences in nasal flora, respiratory allergy burden, and the use of probiotics to intervene in respiratory allergies. Therefore, we summarized relevant studies focusing on occupationally related respiratory allergy in healthcare workers in an integrated narrative review. As this is a narrative review, it was not necessary to record literature searches on specific platforms [23].

## 3. Burden of Respiratory Allergic Diseases Among Healthcare Workers

In China, the prevalence of allergic rhinitis among adults increased from 11.1% in 2005 to 17.6% in 2011, and the rapid increase in the prevalence rate has caused widespread concern, especially in Beijing and Shanghai, where the prevalence rate of allergic rhinitis among adults was higher than 20% in 2011, which is much higher than the average of all cities in China [24]. A 2016 questionnaire study found that the prevalence of chronic rhinosinusitis in Chinese adults was 8%, and the study noted a positive correlation between occupational exposure and the development of chronic rhinosinusitis [25].

Healthcare workers are exposed to a range of allergens such as detergents, disinfectants, natural rubber latex, and a variety of medications in their day-to-day work [26]. Latex gloves can cause allergic rhinitis, asthma, and contact urticaria [27]. Some inhalant allergy principles can lead to chronic respiratory diseases, including asthma and COPD [28]. It has been shown that healthcare workers exposed to allergens are at risk of developing occupationally related allergic rhinitis and asthma (Table 1). In 2016, work-related asthma affected as many as 2.7 million U.S. workers, 10.7–12.4% of whom were healthcare workers, who have one of the highest lifetime asthma rates [29]. In the United States, healthcare expenditures for allergic rhinitis increased by 84% over the five-year period from 2000 to 2005, and 30% of patients miss work because of allergic rhinitis; the prevalence of asthma in the United States was 7.8% in 2008 [30]. Healthcare spending increased from USD 53 billion in 2002 to USD 56 billion in 2007, costing approximately USD 3259 per patient per year [26]. As the severity of asthma increases, the cost of treating asthma gradually increases, and as healthcare workers spend most of their lives at their workplaces, continued occupational exposure will exacerbate the severity of their allergies, which is a huge detriment to their health and undoubtedly imposes an extreme financial burden on healthcare workers.

## 4. Changes in Respiratory Flora in People with Respiratory Allergic Diseases

**Figure 2:**
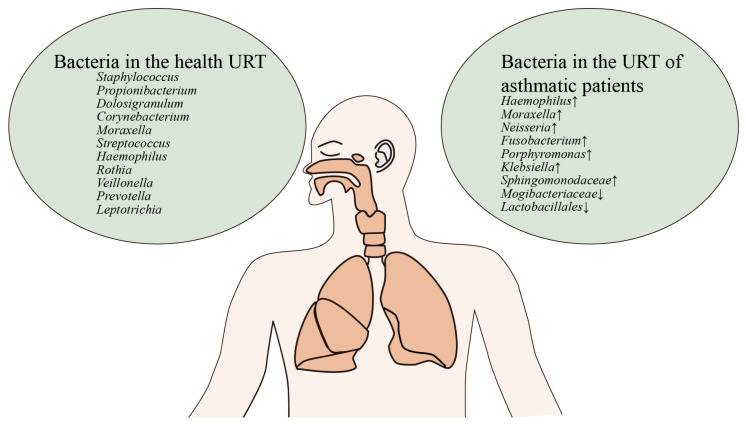
Differences in the composition of the upper respiratory tract flora in asthmatics and healthy individuals. In the upper airways of healthy individuals, all genera were at normal levels; in the upper airways of asthmatics, *Haemophilus* and *Moraxella* were elevated, whereas the levels of commensal bacteria were reduced, such as *Mogibacteriaceae*.

Recent studies have gradually pointed to a strong association between nasopharyngeal flora and the development of allergic respiratory diseases [19]. Significant changes in the nasal flora of patients with allergic rhinitis and allergic asthma during the high allergy season lead to increased inflammatory symptoms in the nasal cavity (Figure 2), while the rise in nasal eosinophils suggests a close link between the inflammatory response to allergic rhinitis and the dysbiosis of the nasal flora [40].

In addition to the occupational allergens mentioned earlier, the adherence and colonization of the nasal mucosa by specific opportunistic pathogenic bacteria is an important factor in the induction of chronic airway inflammation leading to allergic respiratory diseases [41]. For example, nasopharyngeal *Bordetella pertussis* colonization infections are harmless, whereas subclinical *Bordetella pertussis* infections are an important cause of asthma and allergic diseases [42]. Some studies have confirmed that common opportunistic pathogens in the nasal cavity, such as *Streptococcus pneumoniae* (*S. pneumoniae*), *Haemophilus influenzae* (*H. influenzae*), *Streptococcus catarrhalis*, and *Staphylococcus aureus* (*S. aureus*) are positively associated with the onset and exacerbation of asthma symptoms [43,44,45]. Large differences were found by 16S rRNA sequencing between the composition of the nasal microbiota of severe asthma patients, non-severe asthma patients, and healthy individuals, with asthma patients being enriched in *Prevotella*, *Alkanindiges*, and *Gardnerella*. Compared with controls, the abundance of *Prevotella buccalis* and *Gardnerella vaginalis* was correlated with the degree of asthma [46]. Population-based surveys have found a higher abundance of *Streptococcus salivarius* (*S. salivarius*) in the nasal cavity of patients with allergic rhinitis than in normal subjects, and ex vivo models of allergic rhinitis have revealed that *S. salivarius*, a commensal bacterium, alters the morphology of nasal epithelial cells, promotes the release of inflammatory cytokines, and contributes to the development of allergic rhinitis [47]. The researchers also found higher levels of *Staphylococcus epidermidis* and *S. aureus* colonization in the nasal mucus of patients with allergic rhinitis compared to the healthy population, and further experiments revealed that *S. aureus* inhibited the production of IL-33 by epithelial cells and reduced Th2 cell-mediated allergic rhinitis [48].

Occupational exposures have a significant impact on the composition of an individual’s microbiota, and there has been much interest in the concept of the “WORKbiota”, a term which has been used to describe the impact of occupational exposure and work on human microbiota composition [38]. A variety of factors related to occupation have a large impact on microbiota composition, including specific biohazards, circadian rhythms, dietary habits, and work stress [49]. Specific mechanisms of action remain to be discovered by more research. However, some studies have found significant differences in the diversity and structure of gut flora between healthcare workers and non-healthcare workers, and short-term contract workers have higher flora diversity than long-term contract workers, with *Bacteroides* and *Prevotella* being less common in healthcare workers and *Firmicutes* being more common. In addition, flora testing of healthcare workers from different hospital departments and job positions, both short-term and long-term healthcare workers, observed significant differences in the composition of the flora between different hospital departments as well as between individuals with different job positions and different job descriptions. Compared to non-ICU workers, it was observed that ICU workers showed a significant increase in the abundance of *Enterobacteriaceae*, *Phascolarctobacterium*, *Pseudomonas*, and *Streptococcus*, and a marked decrease in *Faecalibacterium* and *Coprococcus bacteria* [50]. Due to the special nature of the work of healthcare workers, their nasal cavity is exposed to various opportunistic pathogens in the working environment for a long period of time, and the composition of the nasal flora changes, which in turn affects their tolerance or susceptibility to respiratory diseases (Table 2). It has been reported that the detection rate of *S. pneumoniae* and *H. influenzae* in the oropharyngeal swabs of healthcare workers in hospitals is twice as high as that of non-healthcare workers [51,52]. A study in Guangzhou noted that among medical laboratory and hospital workers, microbiology laboratory workers had a higher detection rate of *S. aureus* in their nasopharyngeal swabs and that the *S. aureus* detected was more virulent [53]. This may have something to do with the fact that they are frequently exposed to pathogens and are highly likely to spread to family members and partners. Nasopharyngeal swabs from healthcare workers were also found to screen for more drug-resistant *S. aureus* than those from non-healthcare workers [54]. Healthcare workers may bring multi-drug-resistant *S. aureus* into healthcare facilities [55,56]. This has led to an increase in the use of antibiotics, which will pose a new threat in terms of pathogenicity and epidemiology. A high proportion of multiple opportunistic pathogens were also detected on the mobile phones of healthcare workers, where multi-drug-resistant strains were also present [57]. Other studies have shown seasonal variations in the diversity of nasal flora in healthcare workers, with the diversity, abundance, and evenness of nasal flora peaking in autumn [58].

As the first line of defense of the human respiratory tract, maintaining a balanced nasal flora can prevent the respiratory tract from being adhered to and colonized by opportunistic pathogenic bacteria and pathogens, and produce anti-inflammatory immunomodulatory factors, thereby preventing allergen-induced immunoglobulin E (IgE) antibody-mediated inflammation in the airways and autoimmune diseases [68,69]. Thus, respiratory flora may hold the new key to treating allergic respiratory diseases.

## 5. Flora Intervention to Assist in the Treatment of Respiratory Allergic Diseases

**Figure 3:**
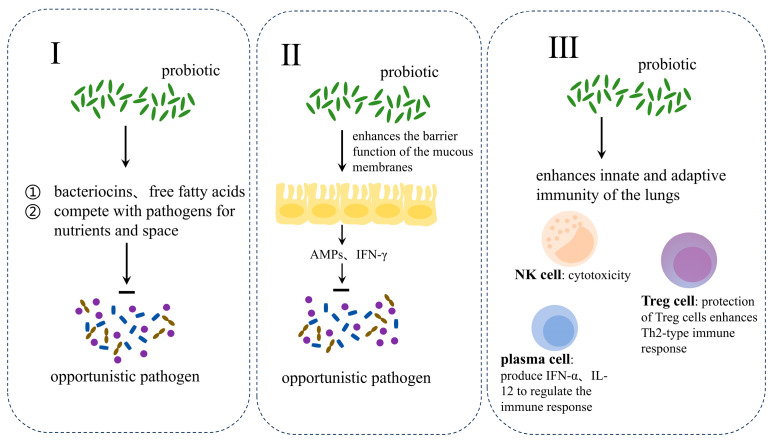
Mechanisms of probiotic-assisted treatment of respiratory allergy. Firstly, probiotics directly compete with pathogens for nutrients and space and inhibit pathogen colonization of epithelial cells. Secondly, it regulates mucosal immunity and promotes secretion of antimicrobial peptides and interferon by immune cells. Thirdly, it enhances innate and adaptive immunity in the lungs.

Current immunomodulatory treatments for asthma are primarily for type 2 inflammatory asthma, such as corticosteroids and antibodies against specific cytokines, but are ineffective in patients without significant type 2 inflammatory asthma symptoms [70]. The most common medications used to treat allergic rhinitis include oral, intranasal, or intraocular antihistamines and intranasal corticosteroids [9]. However, some patients experience adverse effects such as cardiotoxicity, CNS depression, and anticholinergic effects [71]. There is evidence that commensal bacteria in the respiratory tract help to prevent pathogens from adhering to, colonizing, and spreading on respiratory mucosal surfaces [72]. Probiotics are microorganisms derived from human, animal, and plant bodies. The World Health Organization defines probiotics as “active microorganisms that, when ingested in sufficient amounts, produce health benefits for the host” [73,74]. There are some strains of probiotics have been shown to prevent and ameliorate allergic diseases such as atopic dermatitis [75]. Therefore, probiotic therapy offers a promising strategy for healthcare workers to prevent and treat respiratory allergic diseases such as asthma and allergic rhinitis (Figure 3).

### 5.1. Asthma

In animal studies, oral administration of several *Lactobacillus* species showed effective preventive effects against asthma, such as *Lactobacillus rhamnosus* GG(LGG), *Lactobacillus paracasei* (*L. paracasei*) HB89, and *Lactobacillus salivarius* (*L. salivarius*) [76,77,78]. In clinical practice, oral administration of some *Lactobacillus* species has shown beneficial effects in the treatment of asthma in children, such as LGG, *L. paracasei*, and *Lactobacillus fermentum* (*L. fermentum*) [79,80]. In early childhood, lack of exposure to pathogens leads to a shift from a Th1 immune pattern to a Th2 immune-allergic response, increasing susceptibility to allergic disease, and moderate amounts of probiotics can modulate mucosal immune responses and stimulate the innate and adaptive immune system [81]. Thus, oral administration of the probiotic *Lactobacillus* early in life may play an important role in preventing the development of allergic asthma [82]. This is a promising strategy to protect the children of healthcare workers from respiratory allergic diseases.

Probiotics control airway inflammatory response and improve symptoms of allergic asthma by regulating Th1/Th2 cell balance. Animal experiments revealed that supplementation of *L. paracasei* K47 to ovalbumin-sensitized mice can significantly reduce the levels of total IgE and ovalbumin-specific IgE in the serum of mice by regulating the production of Th1 cytokines, thus improving airway hyperresponsiveness and inflammation in allergic asthma [83]. Treg cells play an important role in the prevention of allergic asthma [84]. Smaller numbers of functional Treg cells enhance Th2-type immune responses, leading to eosinophilic airway inflammation. Prebiotics, such as inulin and soluble fiber, produced by the fermentation of *Lactobacillus*, act as a protective agent for Treg cells, thus preventing allergic asthma [85].

Probiotics protect cell proliferation and migration and regulate protein synthesis and gene expression through their bacterial components and secreted substances such as outer membrane vesicles [86]. Intranasal administration of *Escherichia coli* O83 (*E. coli* O83) to mice sensitized to ovalbumin inhibits airway hyperresponsiveness and reduces sensitization-induced respiratory eosinophilia, which is attributed to the activation of the immune system by lipopolysaccharide components in the outer membrane of *E. coli* O83 through the toll-like receptor 4 and the secretion of anti-inflammatory cytokines to inhibit the allergic response [87]. The outer membrane vesicles secreted by *E. coli* O83 (EcO83-OMV) contain the same proteins as those of the parental bacterium in addition to a large number of flagellin and lipopolysaccharides, and it was found that EcO83-OMV could activate the cell-surface receptors TLR2, TLR4, and TLR5, as well as the intracellular receptors NOD1 and NOD2, activate the NF-κB signaling pathway, and promote the expression of downstream target genes such as IFN-γ [88]. And IFN-γ inhibits the production of IL-4, thus indirectly reducing the amount of IgE production, to achieve the purpose of regulating innate immunity to prevent the occurrence of allergic asthma. *L. paracasei* can reduce airway resistance and inflammation in asthma. The extracellular vesicles secreted by *L. paracasei* enhance the phosphorylation of the JNK signaling pathway, which leads to an increase in IL-8 secretion, and achieves the effect of inhibiting airway inflammation in asthma [89].

### 5.2. Allergic Rhinitis

In animal studies, it was found that oral administration of *Lactobacillus* can reduce allergic rhinitis; for example, oral administration of LGG, *Lactobacillus gasseri* TMC0356, and *Lactobacillus plantarum* CJLP133 and CJLP243 can improve the symptoms of allergic rhinitis [90,91]. In the clinic, *Lactobacillus gasseri* KS-13, *Lactobacillus casei* Shirota, and *Lactobacillus acidophilus* strain L-92 have been applied to prevent seasonal allergic rhinitis [92,93,94]. During the pollen season, patients with allergic rhinitis were treated with a probiotic mixture consisting of *Lactobacillus rhamnosus* (*L. rhamnosus*) HN001, *Lactobacillus acidophilus* NCFM, *Bifidobacterium lactis* Bi-07, *L. paracasei* LPC-37, and *Lactobacillus royalei* LE16, which alleviated the symptoms of the allergic rhinitis in the patients after a 2-month treatment, and the study also found that high-mobility group protein N2 (HMGN2) and histone H1.2 may be the target of probiotic intervention in the treatment of seasonal allergic rhinitis [95].

Probiotics improve allergic rhinitis by regulating the inflammatory response indirectly through modulation of Th1 and Th2 cytokine secretion and Th1/Th2 balance. Probiotic NVP-1703, a mixture of *Bifidobacterium longum* and *Lactobacillus plantarum* (*L. plantarum*), was used for the treatment of allergic rhinitis for four weeks and showed a significant decrease in serum-specific IgE compared to the placebo group, while IL-10 levels were maintained at a stable level [96]. Since IL-10 and TGF-β antagonize the biological functions of Th2 cytokines, maintaining IL-10 at relatively stable levels is important for the alleviation and treatment of allergic rhinitis. The Th1 cytokines IFN-γ and IL-12 have been shown to inhibit airway hyperresponsiveness, and *Lacticaseibacillus paracasei* GM-080 induced the production of high levels of IFN-γ and IL-12 by mouse splenocytes sensitized to ovalbumin, thereby alleviating allergic rhinitis in mice [97]. Genomic analysis of GM-080 revealed that its genome contained immunosuppressive motifs (IMs) and CpG-containing oligonucleotides, and that IMs may ameliorate allergic rhinitis through their anti-inflammatory activity, whereas CpG-containing oligonucleotides (ODNs) activate toll-like receptor 9 to shift the immune response from a Th2-type to a Th1-type, contributing to the alleviation of allergic rhinitis [98].

*L. plantarum* GUANKE is a probiotic isolated from the feces of healthy individuals, and previous studies have found that GUANKE enhances SARS-CoV-2 specific immune responses by enhancing interferon-signaling and -inhibiting apoptotic and inflammatory pathways. A more recent study has found that after 4 weeks of GUANKE treatment in patients with allergic rhinitis, a total of 20 serum inflammation-associated proteins were significantly changed, including inflammatory cytokines IL-4, IL-7, IL-20, and IL-33, chemokines such as CXCL1, CXCL5, and CXCL6, and other cytokines such as TGF-α, LAP-TGF-β-1, MMP-1, and MMP-10. This study shows that GUANKE can maintain the Th1/Th2 balance by regulating the functions of various cytokines and chemokines, and therefore, *L. plantarum* GUANKE can be used as an effective probiotic preparation to alleviate the symptoms of allergic rhinitis [99]. *Bifidobacterium longum* IM55 and *L. plantarum* IM76, isolated from feces and kimchi of healthy individuals, were used to treat ovalbumin-sensitized mice, which showed significant reductions in levels of IL-4 and IL-5 and increased levels of IL-10 in the bronchoalveolar lavage fluid, suggesting that IM55 and IM76 can alleviate by restoring the balance of Treg cells and Th1/Th2 allergic rhinitis [100].

## 6. Safety and Efficacy of Probiotic-Assisted Treatment of Respiratory Allergic Diseases

At present, the probiotics used in clinical practice mainly include *Bifidobacterium*, *Lactobacillus*, *S. salivarius*, etc. For different diseases, clinicians will choose different types of probiotics for treatment, such as digestive diseases often using *Bifidobacterium* or *Lactobacillus* for treatment [101]. In contrast, respiratory and oral diseases are often treated with *S. salivarius* [102].

Currently, many clinical trials are demonstrating the safety and efficacy of probiotics (Table 3). In 2020, researchers recruited 200 healthy healthcare workers in Wuhan, China, who were between the ages of 20 and 65 and in close contact with COVID-19 patients, and randomly divided them into an intervention group and a control group, which were given a 30-day prophylaxis with the upper respiratory tract probiotic *S. salivarius*. The results of the study found a significant 64.8% reduction in the incidence of respiratory infections in the intervention group compared to the control group, and a trend toward a decrease in the incidence of respiratory infection symptoms, including an 80.6% decrease in the incidence of low-grade fever. In addition, the researchers observed a 78% reduction in the duration of respiratory infection symptoms in the intervention group, significantly reducing healthcare worker absences and the use of antibiotics and antiviral medications. The study used respiratory infection symptoms, including cough and fever, as observation indicators. Although the subjects were healthcare workers with specialized medical skills who were able to accurately express and diagnose their symptoms, serological diagnosis such as cytokine testing was not performed to accurately diagnose respiratory infections due to the busy schedules of the healthcare workers, which is a major limitation in this study. In addition, the study did not perform 16SrRNA sequencing of the subjects’ nasopharyngeal flora to determine the colonization of *S. salivarius* and the changes in the nasopharyngeal flora of the healthcare workers before and after the flora intervention [103].

Another study also used the probiotic *S. salivarius*. The investigators evaluated the safety and efficacy of probiotics as a supplemental therapy to standard medication for children with recurrent respiratory infections during the cold season and found that supplemental intake of upper respiratory tract probiotics was effective in reducing the morbidity and shortening the onset of acute and recurrent respiratory infections in children as compared to standard medication alone. The results showed that supplemental intake of upper respiratory probiotics was effective in reducing the incidence of acute and recurrent respiratory tract infections, shortening the duration of respiratory tract infections, and reducing the use of antibiotics and antivirals compared with standard drug therapy alone [102].

The safety and efficacy of probiotics for the adjunctive treatment of diseases have been proven in numerous clinical trials, but the public still has certain concerns about probiotics, such as strain variety, probiotic product quality, and dosage of use. The results of a meta-analysis showed that probiotics significantly alleviated the symptoms of allergic rhinitis and increased the Th1/Th2 ratio, and probiotics seem to work as a supplement to improve the quality of life of patients with allergic rhinitis, but after subgroup analyses, it was found that there was a high degree of heterogeneity in the results, and therefore, caution should be exercised when choosing probiotics as supplements for allergic rhinitis [112].

## 7. Discussion

A growing body of evidence suggests a close relationship between human micro-ecology and the development of allergies, including respiratory flora and respiratory allergies, skin microbiota and atopic dermatitis, and intestinal microbiota and food allergies, making flora a target for amelioration of allergic reactions. Probiotics play an immunomodulatory role by helping to establish a healthy microbiota, and they also play a protective role by competing with pathogenic bacteria, maintaining mucosal barrier integrity, and preventing antigen sensitization [113]. The probiotics that have been extensively studied are *L. rhamnosus* and *Bifidobacteria* [114,115]. Although probiotics have been found to modulate the body’s immune system and promote a shift from a Th1 to a Th2 response, the specific mechanisms of probiotics in the prevention of allergies are not known.

Results from mostly randomized controlled trials have shown that probiotics can reduce the incidence of asthma and allergic rhinitis, but due to inter-individual variability, more ex vivo and in vivo trials are still needed to evaluate the safety of all probiotic strains, including chronic toxicity studies. When patients with asthma or allergic rhinitis are immunocompromised, such as infants, pregnant women, patients with underlying diseases, and patients with autoimmune disorders, probiotics may cause them to develop a number of side effects including systemic infections, gene transfer, deleterious metabolic activities, and excessive immune stimulation.

In addition, probiotics may undergo genetic mutations including drug resistance genes and virulence genes under stress conditions, and this effect may alter the original functional properties of probiotics. And most of the current experiments have not examined the stress response of probiotics [116]. Currently, probiotic resistance is relatively understudied, and resistant probiotics may transfer resistance genes to commensals and human-carried pathogens, exacerbating the clinical resistance problem [117]. Future experiments could investigate the stress response of probiotics and the mechanism of action of active ingredients, and personalized probiotic prophylaxis or treatment based on individual differences, which would benefit patients with other occupationally related allergies, including healthcare workers.

## 8. Conclusions

In summary, there is a close link between nasopharyngeal flora and respiratory allergy, and nasopharyngeal flora is a target for the prevention and treatment of respiratory allergies in healthcare workers. Although there is no method to eradicate allergies, probiotics can regulate the body’s immunity and prevent pathogens from inducing the onset and exacerbation of asthma and allergic rhinitis. The use of upper respiratory tract probiotics to intervene in the respiratory flora to prevent and assist in the treatment of occupational allergic respiratory diseases in healthcare workers may reduce the use of antibiotics and inhaled corticosteroids and provide some insights for the future treatment of other occupationally related allergies. However, further studies are needed to determine the inter-individual differences in probiotics.

## Figures and Tables

**Table 1:** Evidence of higher risk of occupationally related rhinitis and asthma in healthcare workers compared to non-healthcare workers.

Year	Regions	Conclusions	References
2007	Brazil	Healthcare workers are more likely to experience asthma or allergic rhinitis symptoms from exposure to latex gloves than non-healthcare workers.	[31]
2013	European	Healthcare workers have 2 times the risk of developing asthma and 1.97 times the risk of developing non-infectious rhinitis than the general population.	[32]
2013	Sweden	The prevalence of asthma among healthcare workers was 4.3%, which was 1.3% higher than among non-healthcare workers and represented an increase in the number of healthcare workers with asthma symptoms compared to the previous year.	[33]
2014	Sweden	Nurses with allergies have an increased prevalence of lower respiratory symptoms, asthma, and allergic rhinitis.	[34]
2016	China	The prevalence of chronic sinusitis in Chinese adults is 8% and is strongly associated with occupational and environmental exposures.	[25]
2016	America	Occupational allergic rhinitis affects 10–60% of healthcare workers.	[26]
2016	America	Chronic respiratory diseases are the most common occupational diseases.	[6]
2020	America Canada	Occupational use of high levels of disinfectants increases asthma rates among nurses.	[35]
2020	Japan	The prevalence of occupational asthma among nurses in Japan is as high as 10.7%.	[36]
2020	Pakistan	The prevalence of allergic rhinitis among healthcare workers was as high as 19.2%, of which 35.9% of working hours were affected by allergic rhinitis.	[37]
2022	European	Occupational factors can interact with bacterial biorhythms and may contribute to the development of disease, leading to the concept of “WORKbiota”.	[38]
2023	England	In total, 12.2% of healthcare workers have chronic asthma.	[39]

**Table 2:** Evidence that healthcare workers carry more pathogenic bacteria and are more virulent than non-healthcare workers.

Year	Regions	Conclusions	References
2014	Sao Tome and Principe	*S. aureus* colonization in the nasopharynx of healthcare workers was 20.5%, more frequent than in patients.	[59]
2015	America	Healthcare workers’ gloves and overalls are often contaminated with MRSA and vancomycin-resistant enterococci (VRE).	[60]
2016	Greece	*S. aureus* is six times more likely to be detected on the hands of medical and surgical staff than in neonatal units, and many of these strains are multi-drug resistant.	[61]
2017	Iran	MRSA detection rate in nasopharyngeal swabs was higher among healthcare workers without any history of risk factors for MRSA acquisition.	[54]
2018	China	Nasal flora of those who perform microbiology experiments have a high detection rate of *S. aureus* and high virulence, which may be transmitted to cohabiting family members or partners; family members of healthcare workers are at increased risk of detection.	[53]
2018	China	The detection rate of *S. aureus* in the pharyngeal swabs of healthcare workers was highest among surgeons (32.4%), followed by nurses (30.8%), and the highest percentage of MRSA was detected in the pharyngeal swabs of nurses.	[62]
2020	India	Oropharyngeal flora of healthcare workers can detect up to twice as many *S. pneumoniae* and *H. influenzae* compared to non-healthcare workers.	[51]
2021	Ireland	Colonization of 4.6% of the nasal and oral cavity by MRSA among healthcare workers.	[63]
2021	Palestine	Cell phones of healthcare workers carry more bacterial species and more drug-resistant bacteria than cell phones of non-healthcare workers.	[64]
2022	Ireland	Methicillin-susceptible *Staphylococcus aureus* (MSSA) carriage was higher among healthcare workers, with 21.5% of them showing both nasal and oral carriage.	[65]
2022	Peru	High levels of resistance to cephalosporins, quinolones, co-trimoxazole, and colistin in *E. coli* detected in healthcare workers’ work clothes and cell phones.	[66]
2022	Cyprus	Multiple multidrug-resistant bacteria, including VRE, MRSA, extended spectrum β-lactamase (ESBL)-producing bacteria, and carbapenem-resistant *Acinetobacter baumannii*, are present in healthcare workers’ work clothes.	[18]
2024	Germany	The prevalence of MRSA among healthcare workers is 1%, while the prevalence of MSSA is as high as 43.7%.	[67]

**Table 3:** Safety and efficacy study of probiotic-assisted treatment of asthma and allergic rhinitis.

Year	Regions	Subject	Probiotics	Results of the Study	References
2010	Germany	Children aged 6–24 months with at least two wheezing episodes and a family history of first-degree atopic disease (n = 131), they were randomized into intervention and control groups	LGG	After 6 months of LGG treatment and 6 months of follow-up, sensitization of infants to airborne allergens was reduced and well tolerated without serious adverse events.	[104]
2017	America	Newborns with at least one parent with a history of asthma (n = 184), they were randomized into intervention and control groups	LGG	The cumulative prevalence of asthma at age 5 years was 9.7% in the intervention group using LGG in the first 6 months of life, compared with 17.4% in the control group.	[105]
2018	China	Adolescents aged 6–18 years with a history of intermittent to moderate persistent asthma for at least one year (n = 160), they were randomized into LP group (n = 40), LF group (n = 40), LP + LF (n = 40), placebo group (n = 40)	*L. paracasei* GMNL-133 (LP), *L. fermentum* GM-090 (LF)	Significant improvement in asthma severity and Childhood Asthma Control Test (C-ACT) scores in the LP, LF, and LP + LF groups compared to the placebo group.	[80]
2022	Meta-Analysis	Children, adults, or the elderly in the community, care facilities, schools, or hospitals (n = 6950)	*L. plantarum* HEAL9, *L. paracasei*	Probiotics may reduce the incidence rate of acute URTIs by about 18%; may reduce the mean duration of an episode of acute URTIs by about 1.22 days. Adverse events from probiotics were minor, and most commonly gastrointestinal symptoms, such as vomiting, flatulence, diarrhea, and bowel pain.	[106]
2022	Meta-Analysis	Pediatric patients with allergic rhinitis (n = 2644), including intervention groups (n = 1362) and control groups (n = 1282)	*Bifidobacterium*, *Saccharomyces boulardii*, *Lactobacillus*, *S. thermophilus*, *Bacillus* and *Enterococcus faecium*	Probiotics improved the remission rate of nasal symptoms, and reduced the serum levels of interleukin-4 (IL-4), IL-6, and IL-17, and significantly elevated the serum levels of interferon -γ and IL-10. Probiotics also reduced the duration of cetirizine use in pediatric AR.	[107]
2023	Meta-Analysis	Patients with allergic rhinitis (n = 3634)	Probiotics, prebiotics, and synbiotics	Gastrointestinal microbiome supplementation (GMS) yielded acceptable benefits for patients with AR compared with controls with sound certainties, after balancing the benefits and harms.	[108]
2023	America Italy Russia	COVID-19 infected individuals who are symptomatic and test positive by COVID-19 (n = 1027)	LGG, *S. thermophilus* DSM 32345, *Bifidobacterium Lactis* LA 304, *L. salivarius* LA 302, etc.	Probiotics supplements probably reduce cough or dyspnea compared to standard care; The probiotic supplement is associated with reduced adverse events.	[109]
2024	China	66 children aged 3–6 years with bronchial asthma (asthma group, n = 66), 35 healthy children undergoing physical examination	*Lactobacillus reuteri* GL-104, *L. paracasei*, *L. rhamnosus*, *Lactobacillus acidophilus* GL-206, and *Bifidobacterium* longum	After probiotic intervention, the abundance of *Bacteroides*, *Clostridium* genera, *Faecalibacterium*, and *Veillonella* in the asthma group approached that of the healthy group.	[110]
2024	Finland	Pregnant women with babies at high risk of allergic diseases (n = 1223), randomized into a probiotic intervention group and a placebo group	LGG, *L. rhamnosus* LC705, *Bifidobacterium* breve Bb99	Probiotic interventions during pregnancy and lactation and altered gut flora in infants and children have a greater impact on the development of allergic rhinitis.	[111]

## Data Availability

No new data were created or analyzed in this study.
